# Comparison of Estimated Late Toxicities between IMPT and IMRT Based on Multivariable NTCP Models for High-Risk Prostate Cancers Treated with Pelvic Nodal Radiation

**DOI:** 10.14338/IJPT-21-00042.1

**Published:** 2022-06-13

**Authors:** Srinivas Chilukuri, Sham Sundar, Kartikeswar Patro, Mayur Sawant, Rangasamy Sivaraman, Manikandan Arjunan, Pankaj Kumar Panda, Dayananda Sharma, Rakesh Jalali

**Affiliations:** Department of Radiation Oncology, Apollo Proton Cancer Centre, Chennai, India

**Keywords:** high-risk prostate cancer, pelvic nodal irradiation, proton therapy, NTCP, late toxicities

## Abstract

**Purpose:**

To compare the late gastrointestinal (GI) and genitourinary toxicities (GU) estimated using multivariable normal tissue complication probability (NTCP) models, between pencil-beam scanning proton beam therapy (PBT) and helical tomotherapy (HT) in patients of high-risk prostate cancers requiring pelvic nodal irradiation (PNI) using moderately hypofractionated regimen.

**Materials and Methods:**

Twelve consecutive patients treated with PBT at our center were replanned with HT using the same planning goals. Six late GI and GU toxicity domains (stool frequency, rectal bleeding, fecal incontinence, dysuria, urinary incontinence, and hematuria) were estimated based on the published multivariable NTCP models. The ΔNTCP (difference in absolute NTCP between HT and PBT plans) for each of the toxicity domains was calculated. A one-sample Kolmogorov-Smirnov test was used to analyze distribution of data, and either a paired *t* test or a Wilcoxon matched-pair signed rank test was used to test statistical significance.

**Results:**

Proton beam therapy and HT plans achieved adequate target coverage. Proton beam therapy plans led to significantly better sparing of bladder, rectum, and bowel bag especially in the intermediate range of 15 to 40 Gy, whereas doses to penile bulb and femoral heads were higher with PBT plans. The average ΔNTCP for grade (G)2 rectal bleeding, fecal incontinence, stool frequency, dysuria, urinary incontinence, and G1 hematuria was 12.17%, 1.67%, 2%, 5.83%, 2.42%, and 3.91%, respectively, favoring PBT plans. The average cumulative ΔNTCP for GI and GU toxicities (ΣΔNTCP) was 16.58% and 11.41%, respectively, favoring PBT. Using a model-based selection threshold of any G2 ΔNTCP >10%, 67% (8 patients) would be eligible for PBT.

**Conclusion:**

Proton beam therapy plans led to superior sparing of organs at risk compared with HT, which translated to lower NTCP for late moderate GI and GU toxicities in patients of prostate cancer treated with PNI. For two-thirds of our patients, the difference in estimated absolute NTCP values between PBT and HT crossed the accepted threshold for minimal clinically important difference.

## Introduction

Elective pelvic nodal irradiation (PNI) in high-risk prostate cancers has been a long-standing controversy [1]. Most international guidelines [2, 3] support elective PNI based on the previously reported prospective and retrospective studies, including a recently published randomized controlled trial (RCT) [4]. However, PNI has been associated with a mild-to-moderate increase in late gastrointestinal (GI) and genitourinary (GU) toxicities as demonstrated by results from the 2 randomized trials incorporating modern hypofractionation regimens [5, 6]. Traditionally, the major focus during prostate radiotherapy planning has been to reduce the higher doses (>65 Gy EQD2) received by normal bladder and rectal mucosa. However, there is now a growing recognition regarding intermediate doses (30-50 Gy) received by bladder, rectum, pelvic musculature, and other substructures impacting the severity of physician-reported late GI and GU toxicities [7–9].

Currently, there are no published RCTs comparing intensity-modulated proton therapy (IMPT) and intensity-modulated radiation therapy (IMRT) for prostate cancers. Retrospective studies comparing these techniques in prostate-only radiation have not shown clinically significant differences either in biochemical control or toxicities [10, 11]. However, most PBT data come from studies using passive scattering technique with or without image guidance compared with photon data, which mostly incorporates modern image-guided IMRT. Although, dosimetric studies comparing these techniques have demonstrated superiority of PBT plans especially with regard to intermediate doses received by bladder, rectum, and small bowel, most of them have evaluated patients not receiving PNI [12–14]. High-risk prostate cancers are also excluded from the 2 ongoing RCTs comparing PBT with IMRT [15, 16]. In the absence of RCTs, a model-based approach has been proposed as a modality for patient selection for PBT [17]. However, this approach has not been attempted for selection of prostate cancers, especially for high-risk prostate cancers.

Our study compares the dosimetry between pencil-beam scanning PBT with that of helical tomotherapy (HT) in patients of high-risk prostate cancers requiring PNI using a moderately hypofractionated regimen. Dose-volume parameters achieved in these comparative plans were used to estimate late toxicities based on multivariable normal tissue complication probability (NTCP) models previously published in the literature [8, 9]. Using the same models, we have also attempted to estimate the percentage of our patients suitable for PBT based on acceptable NTCP thresholds [18].

## Methods and Materials

**Figure 1.**
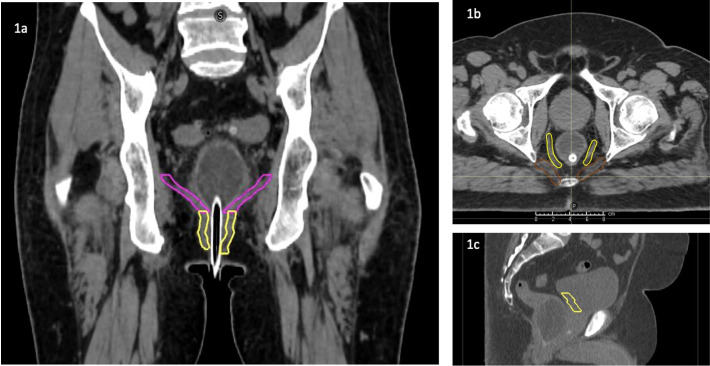
(a) Coronal view of the levator ani complex and external anal sphincter. (b) Axial view showing levator ani and iliococcygeus. (c) Sagittal view showing trigone of urinary bladder.

Clinical and dosimetric data of 12 consecutive patients diagnosed and treated with PBT for high-risk prostate cancers and requiring PNI were included in this study. The study was approved by the institutional ethics committee. The patient images were used to make rival HT plans. A saline-filled endorectal balloon (ERB) was used to immobilize the rectum during the treatment. The entire prostate gland with or without bilateral seminal vesicles was outlined as high-risk clinical target volume (CTV-HR). Pelvic lymph nodes, including bilateral obturator, internal iliac, external iliac, presacral up to S3 level, and common iliac lymph nodes, were defined as low-risk CTV (CTV-LR). Organs at risk (OAR) defined for dose optimization included rectum, bladder, femoral heads, penile bulb, anal canal, and bowel bag. The rectal and bladder wall were defined as the outermost 3 mm of rectum and bladder, respectively [19, 20]. Trigone of urinary bladder, anorectum, external sphincter, iliococcygeus, and levator ani were contoured [19] (**Table 1**, **Figure 1**) to obtain dosimetric parameters for NTCP estimation.

**Table 1.** Delineation of specific organs at risk.

**Organs at risk**	**Cranial**	**Caudal**	**Lateral**	**Comment**
Trigone	VUJ	Urethra	Area between right and left VUJ	Triangular area
External sphincter	2-3 cm above the anal marker	Anal marker	Wraps around the rectum	Cylindrical structure
Iliococcygeus	Posterior edge of ischial spine	Merges with levator ani complex	Between ischial spine and wraps around rectum	V-shaped sling structure
Levator ani	Inner surface of ischial spine	Up to external sphincter	Inner surface of ischial spine	U-shaped sling structure

**Abbreviations:** VUJ, vesico-ureteric junction.

All patients were planned to a dose of 50 Gy in 25 fractions to CTV-LR, with a simultaneous integrated boost of 68 Gy to CTV-HR. The planning target volumes (PTV-HR and PTV-LR) for each of the CTVs were generated using a uniform geometric expansion of 5 mm except posteriorly for CTV-HR, which was expanded to 3 mm toward the rectum. The dose-volume goals to the targets and constraints to OAR for treatment planning (**Table 2**) were per our institutional protocol and were same for HT and PBT plans.

**Table 2.** Target coverage goals and dose constraints for organ at risk.

**Parameter**	**Value (Percentage)**
Target coverage goals	
CTV, all targets	D98 >98% of the prescribed dose
PTV, all targets	D98 >95% of the prescribed dose
OAR, dosage and constraint	
Rectum	V65 <8%
	V60 <15%
	V50 <25%
	V40 <50%
	V30 <75%
Bladder	V65 <8%
	V60 <15%
	V50 <25%
	V40 <40%
	V30 <60%
Bowel bag	V45 <195%
Femoral head	V50 <1%
	V35 <5%
Penile bulb	V50 <50%
	Mean <50 Gy

**Abbreviations:** CTV, clinical target volume; PTV, planning target volume; OAR, organ at risk.

### Proton Beam Therapy Plan

The target and OAR delineation and generation of the proton therapy plan were performed on RayStation treatment planning system (TPS) version 9A (RaySearch Laboratories, Stockholm, Sweden). Two lateral fields (90° and 270°) were used to generate multi-field optimized plans, wherein both fields treated the prostate/seminal vesicles and the relatively central portion of CTV-LR (common iliac/presacral nodes), while each individual field treated the lateralized portion of ipsilateral CTV-LR. In obese patients with skin folds in the beam path due to abdominal sag, a 5*°*/10*°* posterior gantry angle tilt (95*°* and 265*°*) was used to avoid skin folds. All doses for PBT plans were expressed as cobalt gray equivalent (CGE) assuming a uniform radiobiological equivalence (RBE) of 1.1. The spot spacing was set to 1.06 times the average projected sigma multiplied by scaling doctor of 1. Plans were optimized to cover 100% of CTV with the prescribed dose, except at the CTV-rectum interface (at least 95% of prescribed dose). All CTVs were robustly optimized for 5-mm translational errors and 3.5% range uncertainty using minimax robust optimization. Dose calculation was performed for a 3 × 3 × 3-mm grid size. Monte Carlo algorithm (version 4.4) was used for dose optimization and calculation. For proton planning, PTVs were used solely for dose comparison and reporting.

### Helical Tomotherapy Plan

The planning CT and the structure set containing the targets and OAR were exported to Precision TPS (version 2.0.1.1, Accuray Inc, Sunnyvale, California) from RayStation TPS for generating HT plan. Helical tomotherapy plans were optimized to PTV with the same target coverage goals and dose constraints as shown in **Table 2**, using a field width of 2.5 cm, pitch of 0.41, and a modulation factor of 2.0 to minimize the thread effect. These plans were generated using a least squares minimization function for optimization and a convolution-superposition algorithm for dose calculation. All plans were optimized to achieve similar target coverage as achieved by IMPT plans.

### Normal Tissue Complication Probability Estimation

Bladder and rectal toxicities were estimated based on NTCP models published by the University of Groningen (8, 9), using the following **Equation**:



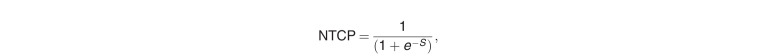

where *S* is a value defined based on the parameters and their respective regression coefficient mentioned in **Table 3** for a specific toxicity. Since the NTCP models were based on conventional dose fractionation, all dose parameters obtained were converted to 2-Gy dose equivalents using the BED formula [21]. Absolute difference in NTCP values between HT and PBT was represented as ΔNTCP for each of the toxicity domains.

**Table 3.** Assumptions for calculation of NTCP.

	**Constant**	**Variables**	**Regression coefficient**
Rectal bleeding (grade II)	−8.09	Anorectum V70	0.3200
		Anticoagulant use	1.1900
Fecal incontinence (grade II)	−7.00	External sphincter V15	0.0640
		Iliococcygeus V55	0.0150
Stool frequency (grade II)	−7.78	Iliococcygeus V45	0.0270
		Levator ani V40	0.0460
Urinary incontinence (grade II)	−9.67	Trigone mean	0.1015
Hematuria (grade I)	−3.45	Bladder wall V75	0.0280
		Anticoagulant use	1.1500
Dysuria (grade II)	−3.87	Trigone V75	0.0210
		TURP	1.0600

**Abbreviations:** NTCP, normal tissue complication probability; TURP, transurethral resection of prostate.

### Statistical Analysis

Dosimetric parameters used for comparison were D95, D98, D2 for PTV-HR; D99, D100 for CTV-HR; D95 for PTV-LR, and D99 for CTV-LR. Incremental doses received by specified volume of urinary bladder and rectum, mean dose of penile bulb, V30 of femoral heads, and V45 of bowel bag were used for dosimetric comparison. One-sample Kolmogorov-Smirnov test was used to analyze distribution of data, and based on that, either a paired *t* test or a Wilcoxon matched-pair signed rank test was used. Statistical analysis was done using IBM SPSS Statistics (version 26, IBM Corp, Armonk, New York).

## Results

### Target Volume Dosimetry

**Table 4** shows the median dose and SD among the 12 patients for various dosimetric parameters for CTV-HR/PTV-HR and CTV-LR/PTV-LR. All PBT and HT plans achieved adequate target coverage satisfying all the pretreatment coverage goals. The difference in dose-coverage parameters between the 2 modalities was not statistically significant except for CTV-D99 (*P* = .00) in the low-risk region and PTV-D95 in the high- (*P* = .016) and low-risk regions (*P* = .00).

**Table 4.** Dosimetric variables noted in helical tomotherapy and IMPT plans.

**Modality**	**Helical tomotherapy**		**Proton therapy (IMPT)**		***P*** **value**
	**Median, Gy**	**SD, cGy**	**Median, Gy**	**SD, cGy**	
High-risk target volume					
PTV D95	67.52	24.43	67.81	31.13	.01
PTV D98	67.03	101.75	67.04	107.49	.31
PTV D2	69.58	50.25	70.21	106.41	.23
CTV D99	67.94	14.25	67.84	13.08	.19
CTV D100	67.37	19.00	67.54	19.68	.19
HI	0.05	0.01	0.04	0.03	.87
Low-risk target volume					
PTV D95	49.49	6.30	49.90	6.50	.00
CTV D99	49.57	5.80	49.94	5.90	.00
	**Median**	**SD**	**Median**	**SD**	***P* value**
OAR					
Penile bulb Dmean (Gy)	28.46	9.51	40.56	10.18	.00
Femoral head Left V30 (%)	7.20	4.94	17.45	9.31	.00
Femoral head Right V30 (%)	7.20	5.30	16.90	12.16	.01
Bowel bag V45 (cm^3^)	130.65	106.84	106.75	98.27	.03

**Abbreviations:** IMPT, intensity-modulated proton therapy; PTV, planning target volume; CTV, clinical target volume; HI, homogeneity index; OAR, organs at risk; V, volume of structure receiving dose.

### Organs at Risk Dosimetry

**Figure 2.**
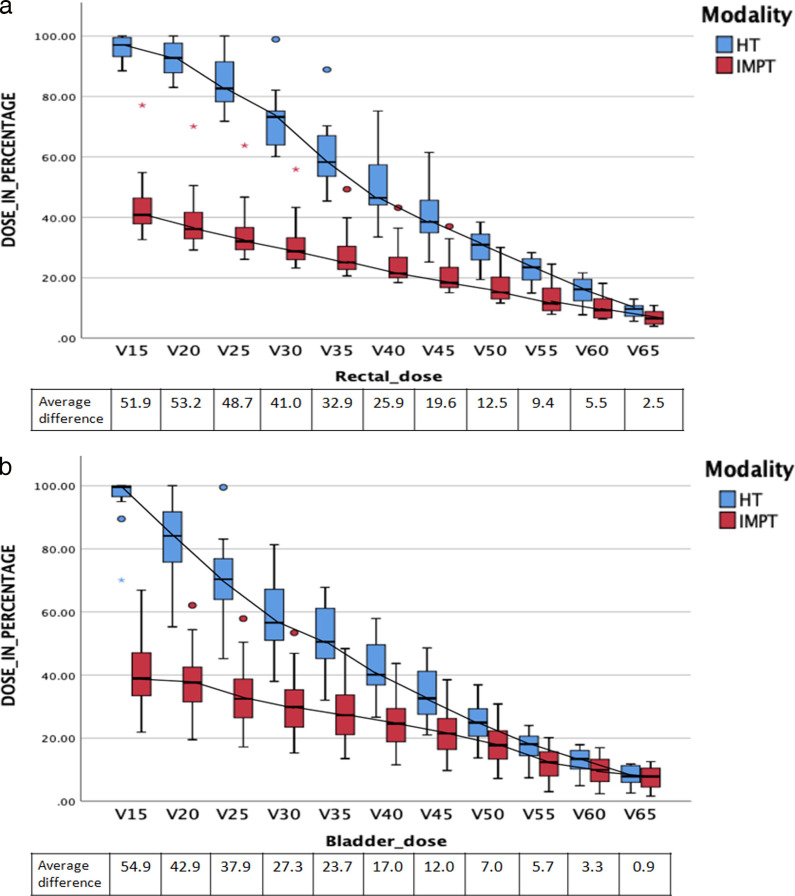
(a) Rectal dosimetry of 12 patients comparing HT and PBT plan (boxplot shows median and interquartile range). (b) Bladder dosimetry of 12 patients comparing HT and PBT plan (boxplot shows median and interquartile range).

**Figure 2a** and **2b** show rectal and bladder dosimetry from V15 to V65 with 5-Gy increments.

The difference in average doses between PBT and HT plans for each of the dose-volume parameters for both bladder and rectum were statistically significant for all dose-volume parameters in favor of PBT. The mean doses received by penile bulb and bilateral femoral heads V30 were significantly higher in PBT plans, whereas V45 for the bowel bag was significantly higher in HT plans as shown in **Table 4**.

### Normal Tissue Complication Probability Comparison

**Figure 3.**
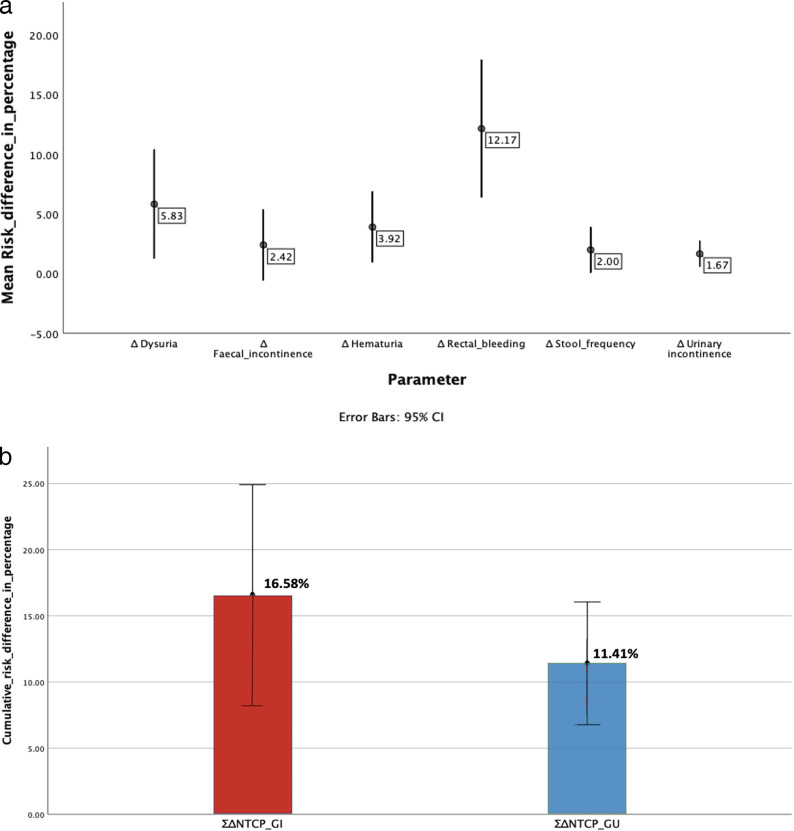
(a) ΔNTCP of each toxicity with mean and error bars showing 95% confidence interval. (b) ΣΔNTCP of each toxicity with mean and error bars showing 95% confidence interval.

**Figure 4.**
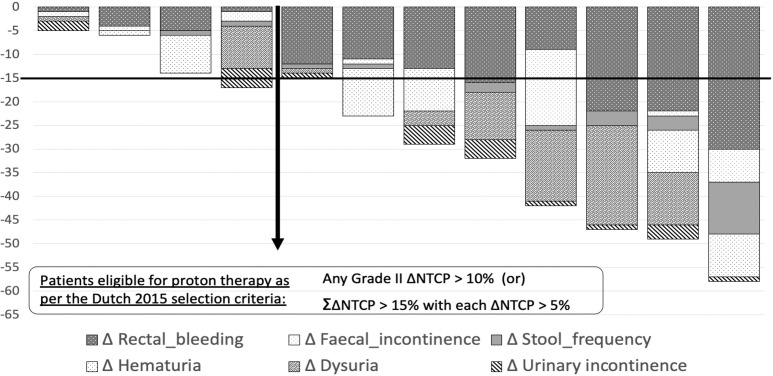
Graph showing ΔNTCP distribution across each patient (arrow separates patients eligible for proton therapy as per accepted criteria).

Among the 12 patients included in this study, 5 had cardiovascular ailments, 4 were on anticoagulants, and 5 had undergone channel transurethral resection of prostate before the treatment. The average risk for rectal bleeding (grade II), fecal incontinence (grade II), and stool frequency (grade II) for PBT and HT plans were 13.75% versus 3.25% (*P* = .002), 2.58% versus 0.17% (*P* = .016), and 2.25% versus 0.25% (*P* = .007), respectively. Similarly, the average risk for dysuria (grade II), urinary incontinence (grade II), and hematuria (grade I) for PBT and HT plans were 15.08% versus 9.25% (*P* = .011), 12% versus 10.33% (*P* = .023), and 8.41% versus 4.5% (*P* = .024), respectively. **Figure 3a** shows ΔNTCP of each toxicity with mean and distribution with 95% confidence interval. The average cumulative ΔNTCP for GI and GU toxicities (ΣΔNTCP) was 16.58% and 11.41%, respectively, favoring PBT (**Figure 3b**). Based on an eligibility threshold for model-based selection, of any G2 ΔNTCP >10% or a ΣΔNTCP >15% with each G2 ΔNTCP >5%, 8 of 12 (67%) patients were found to be eligible (**Figure 4**). With a more stringent criteria of cumulative ΔNTCP >20% and with any G2 ΔNTCP >10%, 7 of the 12 (58%) patients were found to be eligible for PBT.

## Discussion

We compared PBT and HT plans of the initial 12 consecutive patients of high-risk prostate cancers requiring PNI treated at our center. We found that PBT plans led to better sparing of OARs such as bladder, rectum, and bowel bag. There were large differences in rectal and bladder doses between PBT and HT plans in the intermediate dose range between 15 and 40 Gy. To quantify the impact of dosimetric difference on physician-reported toxicity outcomes, we estimated the NTCP using previously published models (8, 9) based on IMRT treatments. Based on the NTCP models used, PBT plans led to a significant reduction in the average risk of G1 hematuria, G2 dysuria, and urinary incontinence; G2 rectal bleeding, stool incontinence and frequency. We also found that based on the estimated NTCP values, two-thirds of our patients would qualify for PBT if the patients were selected using the Dutch consensus PBT eligibility criteria of any G2 ΔNTCP ≥10% or ΣΔNTCP ≥15% with each G2 ΔNTCP ≥5% [18].

Most published photon studies reporting toxicity for patients treated with PNI have shown increased acute GI, late GI, and GU toxicities [22, 23] with a few studies showing no significant differences [24, 25]. The recent randomized studies (PIVOTAL and POP-RT), incorporating contemporary image-guided IMRT schedules and PNI, have also shown either increased late GI or late GU toxicities [5, 6]. The authors of the POP-RT study, which compared prostate-only versus prostate and pelvic RT, hypothesized that increased late GU toxicities noted in the pelvic RT arm could possibly be related to an increase in the intermediate doses (volumes receiving 30-50 Gy) received by the urinary bladder. A similar finding of correlation of G3 GU toxicity with volume of urinary bladder receiving 30 to 40 Gy was observed in a large retrospective study evaluating long-term outcomes of dose-escalated image-guided PBT [26]. Intermediate doses of 30 to 50 Gy to the rectum have also been associated with increased bowel frequency, rectal pain, tenesmus, and fecal incontinence [7]. Proton beam therapy, by reducing the intermediate doses to the OAR, can potentially reduce the above-mentioned late GI and GU toxicities in the setting of PNI.

The potential of PBT to reduce the doses to rectum and bladder were evaluated in previously reported PBT versus IMRT dosimetric comparative studies in patients receiving PNI [27–30]. All these studies noted a significant reduction in the rectal and bladder doses, especially at the low-to-intermediate dose ranges. Similar reductions have been demonstrated in comparative dosimetric studies incorporating prostate-only radiotherapy [12–14, 31]. Unlike other studies, we have used ERB, which improves the setup reproducibility as it ensures stabilization of the prostate during treatment. Also, in the presence of a rectal balloon, the actual delivered doses are likely to be close to the planned doses to target and OAR [32]. Although the doses to rectum and bladder were significantly lower in the PBT plans, the doses to femoral heads were recorded to be higher across all the studies owing to the use of lateral or lateral oblique fields. A similar trend was observed for penile bulb doses in our study, probably owing to a larger lateral penumbra. However, the dose to penile bulb could potentially be reduced if a different beam arrangement, such as posterior or posterior obliques, were used. Despite the relatively small increase in doses to the femoral heads and penile bulb in PBT plans, they were well within the planned dose constraints.

Our study also recorded doses to pelvic musculature, external anal sphincter, anorectum, and trigone of bladder, and they were used to estimate NTCP for late rectal and urinary complications. Most published NTCP models have used older dose regimens, older techniques, and conventional dose fractionation and have estimated higher grade toxicities based on high doses received by OAR [33]. These models are almost exclusively based on prostate-only radiotherapy including the recently published “proton only” NTCP model [34].

Similar to our study, Widesott et al [29] reported an NTCP comparison between helical tomotherapy (HT) and proton therapy in high-risk prostate cancer patients for rectal toxicities using the Lyman-Kutcher-Burman (LKB)-based models. Although they found significant OAR sparing in low and intermediate doses, the NTCP gain was small and insignificant. Their study used LKB-based NTCP models that are based on whole-organ dose rather than doses to specific anatomic substructures [35–38]. Primarily G3 rectal toxicities were estimated by their study, which owing to the advent of modern image guidance are an uncommon phenomenon.

In our study, we have used NTCP models from the University of Groningen [8, 9], which were based on patients treated uniformly with contemporary doses (78 Gy) and modern technique (IMRT) and were multivariable. Although these models were based on prostate-only radiation, they estimated multiple moderate (grade 1-2) toxicity endpoints (G1 hematuria, G2 dysuria, G2 urinary incontinence; and G2 rectal bleeding, G2 stool frequency, G2 fecal incontinence) and demonstrated the impact of doses to several substructures. These models have also incorporated the impact of anticoagulant use and of cardiovascular disease, which have been shown across several studies to impact rectal bleeding and hematuria [39, 40].

The NTCP estimates of the photon plans reported in our study were similar to the toxicities reported in the literature [22–25]. However, the incidence of G2 urinary incontinence noted in our study, while similar to the reported incidence in Schaake et al [9] (12%), is higher than that reported in the literature (<5%). It is possible that the model overestimated the incidence of this toxicity. Although the average risk of all the estimated toxicity domains was significantly lower in PBT plans, the average absolute ΔNTCP for only G2 rectal bleeding and G2 dysuria were more than 5%. However, it should be noted that the average ΔNTCP values can potentially underestimate the benefit of PBT in certain patients. For example, average ΔNTCPs of G2 dysuria and G1 hematuria with PBT were 5.83% and 3.91%, respectively, but 42% of patients had a ΔNTCP ≥9% for both domains.

The model-based selection for PBT has been proposed as an alternative to the standard RCTs. It has been shown that, validated NTCP models for predicting G2 and G3 toxicities in head and neck cancers can be used to select patients for PBT [41] using accepted NTCP thresholds. These thresholds are based on a consensus of Dutch society of radiation oncologists [18]. The same is being contemplated for other sites, such as lung cancers, left-sided breast cancers, and prostate cancers, using similar NTCP thresholds [42]. We have attempted to use the same for our cohort of high-risk prostate cancers. Based on these observed NTCP values, we found that 67% of the patients in our study would be eligible for PBT using a threshold of any G2 ΔNTCP ≥10% or cumulative ΔNTCP >15% with each G2 ΔNTCP >5%. Using a more stringent criteria of cumulative ΔNTCP >20% and with any G2 ΔNTCP >10%, 58% of patients would still be eligible for PBT.

However, this approach has several limitations. Most NTCP models are based on physician-reported toxicities, which are known to be underreported and are based on single institutional experience. Also, most models are based on patients treated with IMRT with conventional dose per fraction. Extrapolation of these models to hypofractionation and for proton therapy may introduce inaccuracies [43]. It has also been seen that with use of variable RBE values, there could be a significant under- or overestimation of toxicities [44]. Since the models used in our study are based on prostate-only RT, they may not have truly captured the impact of reduction in intermediate doses to OAR by PBT. This emphasizes the need for more reliable and long-term prospective or retrospective data of representative cohorts to build robust multivariable NTCP models. These models will also need to be externally validated before they can be used for making clinical decisions on a day-to-day basis [45].

## Conclusion

On dosimetric comparison between HT and pencil-beam scanning PBT for high-risk prostate cancer patients requiring PNI, PBT plans were dosimetrically superior with respect to bladder and rectal doses, especially in the range of 15 to 40 Gy. Based on the dose-volume parameters achieved in this study, PBT plans predicted lower mild-to-moderate GU and GI toxicities compared with HT plans. For two-thirds of our patients, the difference in estimated absolute NTCP values between PBT and HT crossed the accepted threshold for minimal clinically important difference.
